# Understanding success and failure in multimorbidity: protocol for using realist synthesis to identify how social learning and workplace practices can be optimised

**DOI:** 10.1186/2046-4053-2-87

**Published:** 2013-09-25

**Authors:** Sarah Yardley, Elizabeth Cottrell, Joanne Protheroe

**Affiliations:** 1 Primary Care and Health Sciences, Keele University, Keele, Staffs ST5 5BG, UK

**Keywords:** Realist synthesis, Medical education, Health service delivery, Primary care, Socio-cultural theories

## Abstract

**Background:**

Multimorbidity is increasingly prevalent but, aside from epidemiological work, the impact on associated provision of healthcare and/or education is little understood. For example, it is unclear how or why healthcare interventions meet (or do not meet) people’s multiple needs. Professionals working in primary care training sites must reconcile two goals: provision of appropriate individualised healthcare and provision of constructive workplace-based learning for future professionals. Given that professionals, learners and patients may have differing priorities and conceptualisations of success and failure in the absence of cure, achievement of both goals depends on social and cultural mechanisms. This review aims to make sense of how healthcare delivery for, and education about, multimorbidity can be concurrently delivered in primary care through identification of relevant theoretical frameworks.

**Methods/design:**

Realist synthesis identifies and makes sense of variable outcomes caused by interaction between mechanisms and contexts. This review will produce a synthesis of social science, education and primary care literature. Our objective is to understand interactivity between models of workplace-based education and models of patient-centred/integrated care with a focus on perceptions of 'success’ and 'failure’ in multimorbidity. We intend to build a conceptual map and a realist programme theory, populated with evidence from the literature, as the first step towards answering our review question: what is known about how and why concurrent health service delivery and professional medical education interact together to generate outcomes valued by professionals, learners and patients for patients with multimorbidity in primary care? To answer this we are focusing on relationship-based negotiation of needs-based learning and needs-based care as our primary outcome of interest. In this protocol we outline our search strategy and proposed methods of analysis and synthesis of credible and trustworthy data judged to be relevant to our research question.

**Discussion:**

Findings will be submitted for peer-reviewed publication. Identification of how mechanisms of social learning and workplace practices could be optimised to improve quality and utility of patient care in multimorbidity is important. This can inform the future research regarding interventions that will produce a sustainable medical workforce equipped to provide healthcare, when the possibility of cure is absent.

## Background

### Introduction

In 1948 the World Health Organization (WHO) defined 'health’ as 'a state of complete physical, mental and social well-being and not merely the absence of disease or infirmity’ [1]. Contemporary debate highlights that this definition is unsustainable, and possibly counterproductive, in the face of aging populations and changing patterns of disease, with chronic multiple morbidities becoming the norm [2].

In this realist synthesis we aim to develop a (number of) programme theory(ies) to describe how different conceptualisations of success and failure, in the context of health service delivery and workplace-based education regarding multimorbidity arise and influence healthcare/education. The outcome we are focusing on is relationship-based negotiation of needs-based learning and needs-based care. Development of the relevant programme theory(ies) will follow the construction of a conceptual map, detailing existing knowledge about 'what works, for whom, in what circumstances, in what respects, to what extent and why?’ and published understanding of what 'counts’ as success from different perspectives. [3] The results of this review can provide understanding from multiple perspectives to assist endeavours for the continual development (and sustainability through concurrent workplace-based learning opportunities) of a medical workforce equipped to provide healthcare for patients with multimorbidity, when the possibility of cure is absent.

Our focus of interest in the development of a sustainable workforce to deliver needs-based healthcare for primary care patients with multimorbidity was chosen as, other than epidemiological work, there is little understanding of how multimorbidity impacts the work and learning of professionals, postgraduate trainees and medical students. Given that multimorbidity is becoming increasingly prevalent among primary care populations and the co-existence of multiple non-curative problems within individuals is complex and poorly supported by current guidelines, addressing this deficit in current understanding is urgently needed. Achieving this requires enquiry into the interactivity between delivering services to current patients and delivering education to undergraduate students and postgraduate trainees who are becoming the professionals of the future. It is equally important for doctors to maintain their own continuing professional development in both these areas throughout their careers.

We have focused our review by limiting the scope to primary care. This is for pragmatic (a manageable scope) and contextual reasons. Most people with multimorbidity primarily reside outside of hospital (secondary care) settings. Therefore their main interaction with the healthcare system is at the primary care interface. We agree with Barnett et al. that there is an urgent need for strategies effective in 'supporting generalist clinicians to provide personalised comprehensive continuity of care’ [4].

We have specifically focused on education of medical students and postgraduate general practice trainees, although we expect many of our findings will not necessarily be unique to this profession, and we recognise the importance of multidisciplinary working for health service delivery. While acknowledging this, we are limiting our focus to allow us to follow a particular training trajectory from undergraduate to postgraduate to continuing professional education and considering the relationship between this continuum of education and concurrent service delivery for trajectories of disease in multimorbidity.

Primary care training practices are fundamental to two interacting situations that are relevant to this review: direct health service delivery and the training of future professionals. These interventions are complex with interactions arising from a wide variety of relationships between different elements - both agents and structures - and leading to variable consequences including unintended or unpredicted changes in behaviour. General practitioners working in training practices must reconcile two different goals that at times may be either synergistic or antagonistic, that is, the provision of appropriate individualised healthcare and provision of experiential workplace-based learning for future professionals. For example, patients may quite legitimately want experienced professionals to deliver their care but development of experience and expertise is dependent on recognition and acceptance of medical students and postgraduate trainees as legitimate participants in healthcare activities, including holding appropriate responsibilities for patient care.

Given that professionals, learners and patients may have differing priorities and conceptualisations of success and failure in the absence of cure, achievement of relationship-based negotiation of needs-based provision of healthcare and needs-based education in the context of multimorbidity is likely to depend on socio-cultural processes. Programme theories are needed to identify and understand interactions, synergies and tensions between these goals, which will affect the quality and appropriateness of healthcare delivery now and in the future. This understanding of socio-cultural processes will help identify theoretical frameworks, to underpin and inform interventions spanning concurrent clinical and educational goals, which are essential if high quality individualised, sustainable care for people with multimorbidity is to be achieved. The ways and reasons by which agents interact with and adapt to such policies and interventions must be understood if we are to know how 'real world practice’ can be optimised. Both patients and professionals will make meaning and construct knowledge during the trajectories of patients’ lived experiences of multimorbidity. From a socio-cultural perspective these meanings and knowledge constructions are considered to become a part of future interactions [5]. To date, there has apparently been little examination of the issues arising from concurrent health service delivery and training in the context of primary care (or elsewhere) through critique and integration of published literature reporting research in each of these areas. Disease-specific care provision and education carries significant risks of fragmentation of care [6]. Nonetheless, models derived from both theoretical and empirical research for the 'ideal’ delivery of care and the 'ideal’ delivery of education tend to ignore the fact that both occur in the same place, at the same time, involving the same people, and both are affected by how people think, feel and act in relation to each other and the circumstances in which they find themselves. Everyday general practitioners in training practices experience the need to balance health service and workplace-based education agendas but understanding of issues in combining concurrent learner-centred education with patient-centred care in practice has not been adequately researched to date.

In this protocol we provide definitions of our areas of interest and make explicit why this review is important before describing our study objectives and research questions, tentative theories to be tested, and study design including theoretical orientation. A realist synthesis is, by its nature, iterative so this is not a 'protocol’ in the biomedical tradition. We are, however, providing explanation and justification of our methodological reasoning and the methods chosen to address a complex set of research questions.

### Definitions

Multimorbidity has been defined as 'the co-existence of two or more chronic conditions, where one is not necessarily more central than the others’ [7]. For this current work, relevant conditions must: be distinct clinical entities rather than one condition being an extension or direct complication of another, cause patients to experience troublesome symptoms, and currently have no definitive cure (at least for the vast majority of patients). We have not limited our interest to any specific stage of condition trajectories. Examples of co-existing conditions which we consider to fall within this category include: heart failure, diabetes, chronic obstructive airways disease, cancer, neurological degenerative diseases, musculoskeletal diseases and long-term mental health conditions.

The term 'primary care’ can mean different things in different healthcare systems. We are using the term to encompass care led by general practitioners in the UK or the nearest equivalent elsewhere. Hence this includes, for example, family practice in the US but does not include emergency department hospital-based care. By health service delivery we mean any care provided to individuals or groups of patients by qualified health professionals and the associated structures and institutions through which this care is organised.

Experiential workplace-based medical education is defined as authentic involvement in healthcare activities (for example, a general practice surgery, home visit or similar) involving real patients and professionals as well as 'learners’ who may be at any stage of training from undergraduate to postgraduate and including continuing professional education when it is directly linked to learning from experiences.

### Why this review is important

#### Changing demographics of health and illness

It is increasingly difficult to define health and illness in a time of aging populations and new patterns of disease. This is exemplified by the blurring of boundaries between long-term, chronic, progressive and life-limiting illnesses and the emergence of 'survivorship’ as a construct to describe recovery or remission from diseases from which there is still no known definitive and permanent cure (or consensus thereof). Increasing numbers of people experience variable (in both number and intensity) symptoms from multiple clinical diagnoses [7]. To provide a contextual illustration of the size of the problem, a recent epidemiological study of multimorbidity in Scotland (including all adults registered with a general practice, *n* = 1,751,841 in 314 practices) found that over 40% of the population had at least one morbidity from chronic disease, with 23.2% having multimorbidity and 8.3% having at least one physical and one mental health morbidity. By the age of 65 years the majority of people (almost 200,000) registered with a general practice were multimorbid, with those living in deprived areas more likely to experience this at all ages other than in the over 85-year-old group. The authors of this study acknowledge Scotland’s higher rates of socio-economic deprivation and lower life expectancy compared to other developed countries but the trends in their findings have also been replicated elsewhere [4].

#### Response of policy-makers and institutions: proposed ideals

The most common solutions proposed to meet the challenge of multimorbidity are patient-centred care and integrated care [8]. Although patient-centred care has been defined theoretically as care which includes psychological, social and physical needs-based approaches, accounts for patients’ priorities and concerns and is delivered through shared decision-making, patient-physician partnerships and co-ordinated working across healthcare systems [7] less is known about the realities of negotiation of such care in practice. In addition, existing integrated care pathways for specific diseases do not address the problem of multimorbidity [9].

Practice-based implementation of strategic policies for integrated care is challenging as few studies explain how or why healthcare interventions meet (or do not meet) people’s multiple needs. Professionals and patients may have differing priorities and conceptualisations of risk-benefit balance. Transitions between care settings and transitions in care priorities are particularly difficult for professionals and patients to manage. It is currently unclear how the reorganisation of health services within the UK National Health Service, including new systems of commissioning, will impact on these issues. In other countries different healthcare systems are likely to present different challenges, although the underlying issues of system design and structures which may not be flexible or easily adapted are likely to remain. If it is accepted that healthcare should be available to all on the basis of need rather than diagnosis, then equity of access to needs-based care requires a primary care community approach earlier in patient illness trajectories [10].

#### Current trends in clinical practice

To date multimorbidity has mainly been understood using models of an index condition with associated complications and/or additional co-morbidities [7]. This disease-specific approach is subject to criticism on at least two counts. Typically the index condition has been defined by professionals according to biomedical models (or specialty interests) that may fail to account for individual patient perspectives and variance in symptoms or functional impact. This approach also ignores those experiencing multiple 'low-risk’ conditions that, collectively, are important for both the individual and health service demand. Professionals will be ill-equipped to manage multimorbidity if medical education follows traditional disease-specific models of teaching, exemplified in the belief that good clinical reasoning should seek to find a single diagnosis (or at least the minimum number) to explain a patient’s symptoms.

As medical practitioners must care for patients with numerous variable combinations of conditions, a proliferation of guidance that requires them to think and act with respect to a particular index condition has emerged. This guidance is based on evidence and relates to a situation that rarely fits any specific patient’s circumstances or combinations of multimorbidity. Many services are designed to meet current best evidence recommendations, this may result in patients with multimorbidity finding healthcare provision too inflexible or, at worst, inappropriate for their multiple, concurrent needs.

#### Current trends in medical education

Despite the advent of integrated undergraduate curricula and problem-based or case-based learning it is common for students and trainees to learn about one, or a very limited number of conditions at a time. This inadequately prepares professionals to care for patients whose problems or circumstances deviate from specific protocols or care pathways or to make clinical decisions management when 'best practice’ for conditions present within one individual is discordant. Further, the majority of clinical training, even for general practitioners, traditionally, has taken place in hospital settings, thus providing a disproportionate exposure to learners of acute problems, managed in a specialty specific way, rather than a wider focus on holistic, chronic and preventative community-based care.

Better understanding of the complex environments, within which workplace-based experiential learning opportunities are set, is required [11]. It is not possible (even if it was desirable) to simplify the complex workings of clinical practice environments. Credible solutions for offering useful learning opportunities in these contexts must account for inherent uncertainty and unpredictability. The problem of developing generic skills and transferable knowledge has long taxed medical educators [12,13]. Preparedness is currently being questioned as a suitable concept on which to build transferable learning as there is increasing recognition of the importance of context within the field [11,14]. A proposed alternative to seeking to develop generalisable knowledge and skills is to learn to seek to clarify understanding of challenges in context for medical education [11]. Given the clinical problems described above it is necessary for doctors (both those currently qualified and future practitioners) to engage in education and professional development that will equip them to provide appropriate care to patients with multimorbidity throughout trajectories of illness [15]. A significant challenge to contemporary medical practice is the need to promote positive and holistic approaches to patient care in a range of situations where 'cure’ is not an option [7].

#### Why realist synthesis as a review method?

Realist synthesis is a form of theory driven interpretive systematic review method which is designed to study complex interventions in complex systems. Early examples include work to inform public health policy [16]. A realist synthesis seeks to analyse evidence (regardless of nature as long as it is judged to be relevant and of sufficient quality to support the inferences being made) in order to understand interactions between context, mechanisms and outcomes. The approach taken to any area of interest can be summarised as seeking explanations (to some, or all, of the question) of 'what works, for whom, in what circumstances and why?’ [17]. In using this approach we are conceptualising health services delivery and medical education within clinical workplaces as complex social interventions [3]. Whether or not there are existing models for these two activities, with respect to multimorbidity it is important to consider how social context influences the achievement of desirable outcomes for patients and future professionals (and their patients).

## Methods/design

In realist methodology the terminology 'CMO’ refers to 'context, mechanism(s) and outcome(s)’ - a phrase designed to ensure attention is paid to the linkage and relations between these three elements as well as each element itself. As Pawson emphasises, the CMO configuration should not be used to create three disconnected lists of the elements of a programme theory but purposefully seek to look at the function of each part of CMO in relation to the other parts within the programme theory [18]. The 'CMO’ of a particular project defines the shape and content of underlying programme theories which describe something that 'works’ or how a particular consequence/goal is achieved (outcomes) through identification of underlying mechanisms which operate in particular contexts. The choices people make (and other expressions of agency), whether individually or collectively are, in realist terms, important mechanisms for the generation of social outcomes.

One of the challenges regarding primary or secondary synthesis of socio-culturally mediated interventions or processes is to identify the mechanisms by which desirable outcomes are produced. Moving from the abstract to the specifics our work we have identified a need to study how health service delivery and workplace-based education 'work’ with respect to relationship-centred negotiation of needs-based learning/care. To understand this is to understand the mechanisms that need to be triggered to produce our outcomes of interest in the (continually changing) context of primary care. We have explained in the introduction above, and in the study objectives below, our reasons for focusing on the contemporary challenge of multimorbidity and situating our work within the context of primary care. We are tentatively proposing that socio-cultural theories can be used to unmask and identify the mechanisms at play when health service delivery and workplace-based education are occurring concurrently by the same people, in the same place, at the same time. More specifically, we argue that understanding what 'counts’ as success for the different people involved (learners, patients and professionals) will shape their interactions and influence the social and cultural aspects of mechanisms which lead from context to outcomes of relationship-centred negotiations of care and education or not. We believe interactivity is important and are seeking to describe this as an underlying mechanism which may or may not produce desired outcomes.

This protocol has been designed using the recently published standards for realist syntheses [19]. We will also use these standards in reporting our findings. Our study will initially explore the relevance (or not) of Vygotskian theories of workplace learning and social practices to multimorbidity (see section on theoretical orientation below). During later literature searching, and while conducting our synthesis, we anticipate an iterative process, in line with realist methodology, will occur leading to progressive focusing of the review. We intend to follow published advice on how such focusing can appropriately occur [19].

### Study objectives and research questions

Our objective is to understand interactivity between models of experience-based workplace education and models for patient-centred/integrated care as mediated by socio-cultural processes with respect to our initial review question which is: what is known about how and why concurrent health service delivery and professional medical education interact together to generate outcomes valued by professionals, learners and patients for patients with multimorbidity in primary care? To answer this question we are focusing on relationship-based negotiation of needs-based learning and needs-based care as outcomes potentially valued by professionals, learners and patients when dealing with multimorbidity. We have focused on multimorbidity because it is increasingly recognised as one of the greatest healthcare challenges of our time [4].

We will initially approach this question from a socio-cultural perspective (informed by the research team’s own experiences as clinicians, patients and learners), acknowledging that how people define success and failure influences their interaction with other people, organisations or institutions (that is, context and culture matter). Contemporaneous learning about multimorbidity alongside healthcare delivery requires social negotiation of definitions of success and failure. In our synthesis we will explore how relationship-based negotiation of needs-based care and education occur. We believe this is an important first step in this investigation of concurrent medical education and health service delivery because conceptualisations of good medical and educational practice are dependent on the various definitions of success and failure of those involved (that is, meaning influences behaviours, negotiations between patients, learners and professionals, roles and responsibilities, decision-making and clinical judgements, all of which, in turn, will impact on whether needs-based and relationship-based outcomes are achieved). Once we know how and why success and failure are conceptualised in multimorbidity, where there is an absence of cure, and the effect of this on evolution of knowledge, meaning and practice, we can then develop programme theories designed to inform the development of a sustainable, appropriately equipped, workforce that can deliver holistic patient-centred care for this growing population.

The variables and issues we outline in our more detailed initial review questions below may exist within the mechanisms of our very tentative programme theory to explain our outcomes of interest. These are all interconnecting parts of the realities of clinical practice which occurs alongside workplace-based experiential learning in medicine/medical education:

1. How and why does learning about and delivering healthcare in primary care for people with multimorbidity work (that is, what are the programme theory or theories for learning about and for delivering healthcare? These may, of course, overlap and intertwine with each other)? Including more specifically:

a) Which, if any, theories are used in the literature to explain mechanisms for concurrent education and health service delivery in this area?

b) What are the relevant outcomes for patients, teachers and learners and how do these come about?

c) What differences (if any) are there between 'policy’ and 'practice’?

d) What role (if any) do social interactions and (differing) conceptualisations of success and failure play in mechanisms of concurrent education and health service delivery in this area?

2. What areas for further research can be identified to inform the development and sustainability of a medical workforce to provide care for patients with multimorbidity?

### Theoretical orientation

In our review we will draw on realist philosophical principles to build theory and refine understanding of how outcomes occur in both health service delivery and medical education with respect to learning about and treating patients with multimorbidity. The realist perspective on these socio-cultural issues directs us to consider how complex interventions are reliant on people making choices, have long implementation chains, will produce different outcomes in different contexts that are multiple, planned and unplanned and often contested, and also to consider how what has happened previously will shape what happens next and that interventions evolve during implementation making change itself an evolving entity. It is argued that there are seven key considerations about intervention programme complexity: volitions, implementation, contexts, time, outcomes, rivalry and emergence (VICTORE) [18] - all of which may need to be considered with respect to 'unpacking the black box’ of how certain mechanisms might, under certain contexts, produce certain outcomes or not. Exploring these influences from a socio-cultural theoretical orientation (see below) will allow us to begin to unpick influences in the practical workings of concurrent education and health service delivery. In our work we will consider evidence beyond specific interventions if it is about programmes of activities designed to deliver care to or teach students about the care of patients with multimorbidity. We will construct one or more programme theories that explain our outcomes of interest. Based on our scoping searches of the literature, (see below), we think that a good starting point for this is to consider if socio-cultural theories of a Vygotskian tradition have explanatory value for our outcomes of interest.

Vygotskian derived socio-cultural theories, for example, Situated Communities of Practice [20,21] and Activity Theory [22-24], are concerned with bi-directional influences between individual and collective knowledge construction and meaning-making, considering the social culture within which people interact. In a similar manner, goals of individuals and institutions can also be considered in context using socio-cultural approaches. For example, Activity Theory provides a model to consider goal related joint activities of people within complex processes involving both individual and institutional expressions of agency [22-24]. Using these theories is appropriate to our research questions as we are concerned with the pursuit of two concurrent goals within the activity systems of primary care workplaces. There is a common misconception within medicine that the alternative to disease-modifying (often less precisely termed as active treatment) approaches is 'doing nothing’. Context including culture must be accounted for if we are to understand how and why 'good medical practice’ is conceptualised with respect to multimorbidity and the implications of these social constructs for health service delivery and medical education. Therefore, we need to know how and why success and failure are conceptualised in the absence of cure if we are to achieve relationship centred negotiations of needs-based care and a sustainable workforce to deliver this care.

These underpinnings have shaped our research questions and will allow the review to be conducted with the understanding that peoples’ expectations and perceptions can shape their experiences and resultant personal or collective meaning-making and knowledge construction. We are using the realist review method to build theory in this instance, clarifying what does happen rather than what should happen (and why). Understanding what does or does not happen within 'real life’ working practices is important because the purposive social actions of people and institutions are subject to the law of unintended consequences: there is always potential for unanticipated effects [25]. Within our review of the literature we will seek to identify if these theoretical perspectives have been accounted for in interventions or policies designed to address education and/or health service delivery, or if competing theories are drawn on instead, considering the implications of this.

Given that there is no apparent single overarching theory, realist review is being used as a method to identify and evaluate possible, competing theories for our outcomes of interest. In the context of policy implementation Pawson and Tilley define programmes as theories embedded and active within open systems with the intention of bringing about change [26]. In the absence of any deliberate interventions human beings still behave in semi-predictable ways in particular settings that result in outcomes or consequences (incorporating impact as well as specific outcomes), albeit these may be different to those anticipated [25]. The identification of the relationships and configurations between context, mechanism and outcomes are useful to explore and explain the 'real world’ practices and social interactions that occur in multimorbidity care. Used in this way the realist approach can facilitate identification of contexts that are useful or necessary to achieve desirable outcomes, to conceptually map what is known as an initial step in developing programme theory, alongside identifying areas of research need (known unknowns). Indicative examples of the possible elements of context, mechanisms and outcomes relevant to our work are outlined below (section on data extraction).

### Setting

The review is being conducted by a core team including people with the following perspectives and expertise: clinical academic researchers with experience in systematic and narrative reviews, qualitative and quantitative research, medical education, social sciences and general practice; patient and public involvement group members with experience of co-/multimorbidity from chronic conditions, and; undergraduate and postgraduate medical trainees. In addition there are a number of expert advisors providing input into realist synthesis methodology and applied methods, statistical quality assessment of the literature, and translation of non-English language literature. The team is being coordinated by the lead author. All core team members have read and discussed methodological literature for realist synthesis, and several have undertaken specific training on conducting a review with this methodology. The review will focus on the contextual issues of UK healthcare system with respect to education and health service policy while considering potential implications of the work in other settings.

### Details of literature search

We are interested in two possible empirical situations, particularly if these are implemented within the same context concurrently. First, we are seeking empirical studies of any intervention which purports to lead relationship-centred negotiation of needs-based healthcare and education. Second, we are seeking empirical studies of any intervention which purports to provide workplace-based experience in managing the complexities of chronic illness/multimorbidity with primary care settings. For either intervention we will be seeking to understand if interactivity with either concurrent education or service delivery has been considered.

Preliminary searches of the literature suggest that such interventions are likely to be relatively rare (although not non-existent), and concurrence of the two even more so. Rather, it seemed the literature was most likely to reveal the depth and breadth of angst related to the 'social problem’ of multimorbidity from a variety of perspectives. Therefore we have designed the searches detailed below to identify evidence that might populate a conceptual map of what is known and what is unknown with respect to our research questions. This means that we have deliberately not limited the search to interventions so that any information relevant to our research aims and questions can be sought to inform our work. Such avoidance of pre-determining literature for inclusion/exclusion according to methodology, methods or coverage of all elements of a review question is consistent with realist methodology [18]. We will build on our scoping searches as described below. We acknowledge that this is an iterative process, during which we will refine our focus and tailor our searches to ensure we seek additional literature to refine any developing programme theories. Therefore it is not possible to give full details of the final searches until we report our findings. What follows sets out our intentions.

#### Identification of the literature

##### Round 1

Preliminary scoping searches undertaken to shape this protocol used the terms (multimorbid*.ti.ab. OR multi-morbid*.ti.ab.). A systematic search strategy has now been built in Medline and adapted for other databases. We have included a total of 20 databases covering healthcare, education and social sciences. These are: Academic Search Complete, Applied Social Sciences Index and Abstracts, Australian Education Index, British Education Index, Best Evidence Medical Education, British Nursing Index, CINHL, Cochrane, Embase, Education Resources Information Center, Health Management Information Consortium, Joanna Brigg Institute, Kellogg Foundation, Medline, Opengrey, PsychInfo, Science Direct, Social Services Abstracts, Sociological abstracts and Web of Science. In addition we have included in our searches Google Scholar and Web of Science key author searches and emailed identified experts in the field for further suggestions.

The search strategy was built with a combination of four searches. These were designed to capture publications relevant to: (1) multimorbidity; (2) primary care; (3) education; and (4) workplace experiences. The details of the search built for Medline are given as an example in Additional file 1. To focus the literature search on our research questions these four searches have been combined as (1) AND (2) AND ((3) OR (4)). This search was completed in all databases on 1 August 2012 with alerts set up to identify future additions.

During the development of the protocol, round one title screening was also carried out by SY. Articles were only excluded at this stage if clearly not relevant to one of the four search items above. Abstract screening, also carried out by SY, excluded articles which were not about multimorbidity (articles retained if about chronic disease, for example, chronic disease models, but not disease specific, as likely to include multimorbidity) not applicable to primary care (articles retained if about primary care and other settings, or considered to be directly relevant to primary care (that is, same mechanisms may be in operation) despite alternative setting of study). The remaining articles were then read and categorised by SY as pertaining to one or more of: health service delivery, medical education or social processes. Any articles not pertaining to any of these items, but not clearly excluded by the criteria above, were included/excluded after discussion with the other members of the research team reached a consensus to do so. We next purposively selected articles which have been categorised as pertaining to two or more of these areas (*n* = 53) in order to focus on our objective of identifying what is known about how and why concurrent health service delivery and professional medical education function with respect to multimorbidity in primary care and our interests in the role of social processes and interactions. If required, we retain a database of articles only coded as one of the above items from which we can seek further information to inform the development of our conceptual map/programme theory or theories in round 2. We will also extract potentially relevant citations from identified papers for consideration in round 2. The protocol for round 2 is detailed below.

##### Round 2

Additional articles published since August 2012 will be identified for inclusion through our database alerts and reviewed for inclusion according to the same criteria as used in round one. We will also return to those articles identified from round one papers and those identified by the round 1 search which pertained to just one area of health service delivery, medical education or social processes: SY and EC will screen these together for further information to contribute to our synthesis beyond that already identified. SY and EC will also screen potentially relevant policies and reports in the grey literature to inform our work. Examples of these include documents from the WHO, UK Department of Health, National Institute of Clinical Evidence, Commission for Quality Care, King’s Fund, Nuffield Trust, Research Councils, General Medical Council and colleges and organisations for Medicine and Medical Education.

#### Analysis and interpretation

Making sense of a pattern of findings in a realist synthesis is often done by drawing on existing theories, which can be tools to aid critical thinking in the synthesis whether this is to juxtapose, reconcile, consolidate or situate ideas or to adjudicate between studies [27]. Our approach to analysis will involve bi-directional use of theoretical socio-cultural perspectives as described above and empirical literature, using each to critique the other, while the overall process is guided by the principles of realist synthesis: seeking an explanatory focus, incorporating data generated through multiple methods and inter-relating context, mechanisms and outcomes. Our analysis and interpretation will have the potential to ultimately produce two related outputs. First a conceptual map, detailing what is known about conceptualisations of success and failure in the absence of cure and the influence this has on concurrent education and health service delivery now, and with respect to sustainability for the future, as well as the identified 'black holes’ of unknowns. Second, we intend to develop this into a (number of) realist programme theory(ies), using Pawson’s seven key elements of any implementation programme (VICTORE - see above [18]) in addition to the realist model of context, mechanisms and outcomes (CMO). An overview of how we intend to achieve these outputs is given below.

#### Data extraction

Given the complexity of our research questions and objectives we have designed a data extraction sheet (see Additional file 2) for our purposes. In order to identify data that is needed to build a programme theory it is important to capture as much relevant data as possible that will enable us to build a picture of how 'good’ multimorbidity care and education to make this care sustainable is produced. The data extraction sheet has been produced to standardise the focus of each review team member on the 'CMO’ (context, mechanisms and outcomes) configurations within included studies as well as data which might help us to understand and explain: (1) the CMO configurations themselves; (2) the relationships between them; and (3) their role and place in our programme theory or theories. Each included article/item of literature will be independently assessed by two members of the review team for relevance to our research questions and objectives and for rigour. Those items meeting these criteria will then undergo full data extraction by two members of the team working independently. SY will complete data extraction for all articles/items with EC/HC/ER (see acknowledgements) dividing the second extraction between them. Once completed each pair of reviewers will meet to compare their data extractions and reach a consensus on completeness of data extraction and interpretations made with respect to coherence and plausibility. In addition the patient and public involvement group members (AW/AH - see acknowledgements) will be involved in the process with guided data extraction of an arbitrarily selected number of articles followed by those purposively selected as purporting to specifically address patient perspectives. This is to facilitate these group members to understand the review process, which they felt would be useful preparation for discussing potential programme theories as the synthesis progressed. Data extraction will include documentation of any theories explicitly or implicitly referred to whether these are socio-cultural in nature or from competing perspectives. As we identify data to develop our programme theory we will begin to focus more specifically on extracting data that can help us to refine the theory. There is, undoubtedly a process of iteration required to do this. We outline below indicative examples of the type of data that might be needed to help develop our programme theory, cross-referenced to the 'CMO’ (context, mechanisms, outcomes) of realist methodology:

##### Contextual and process data (used to identify 'C’ - context, and infer possible 'M’ mechanisms for testing)

Information about the study design or type of article, identification of patients (trajectory of illness) and learners (stage of education), whose perspectives are represented and any identifiable drivers for interventions. Information about the activities and goals in practice of any intervention for health service delivery and/or workplace-based education including any models or theories used. Information about 'real world’ practices including alterations, fragmentation or changes in focus from design or desirable working practices. Information about social processes and interactions including key areas of interest in socio-cultural theories such as expressions of agency, interactivity, negotiation, taboos, roles and identity, structures and artefacts. Information about the construction of interactions including general attitudes, use of metaphors, causal statements or claims, narratives, constructions of success or failure in the absence of cure, and issues of risk/responsibility/trust. These process data elements are designed to allow us to critique the literature from a socio-cultural perspective and will be subject to modification during the iterative process of the review.

##### Data about consequences (used to identify 'O’ - outcomes and impact and infer 'M’ possible mechanisms)

Information about any intended or unintended outcomes or consequences with respect to provision of healthcare and/or provision of workplace-based learning for professionals. Any information related to evidence of sustainability of health service delivery through engaging in education for future professionals within workplaces delivering healthcare to people with multimorbidity in primary care. In order to further focus on conceptualisations of success or failure in the absence of cure we will also specifically seek to extract words or metaphors that provide insight into this area.

Following the first round of data extraction the whole team will meet to discuss: (1) emerging findings; and (2) strategies to select additional literature sources to include in the review from new literature identified in the review process and/or alternative sources which might further inform the development of our programme theory. Where necessary, to increase our understanding of our initial programme theory (or theories) we will undertake a more 'fine-grained’ realist analysis of context, mechanisms, and outcomes (using the CMO model) of each part of our programme theory. The combination of the realist analytic framework of context, mechanisms and outcomes with our specific focus on interactivity from socio-cultural perspectives will initially guide our interpretations. As the review progresses and our understanding of our topic are grows, we will seek out and draw on any other relevant theories to explain how the various outcomes within our programme theory (or theories) come about. We intend to use mind mapping software to document and develop our interpretative analysis of the literature as a team.

#### Intended outputs

The review will result in methodological insights (arising from the application of this review method to a new area of complexity) and clinical/educational findings including a realist programme theory relevant to the objectives and questions posed above. We anticipate our work, particularly the methods we used, will be helpful for others seeking to find similar materials to address equally complex and concurrent issues in the future in the following ways.

### Management and governance

This protocol has been peer-reviewed within our University institution. It does not require ethical committee approval as it is a realist synthesis (secondary research) of existing literature. We have built a review team which represents diverse perspectives and roles including clinicians in primary and secondary care, academics in medical, social science and educational fields, patients and members of the public, undergraduate medical students and postgraduate medical trainees.

## Discussion

Realist syntheses are not intended to produce a 'right’ answer but understandings of how interventions or other deliberate processes produce diverse effects [28]. We have discussed above how we are using realist logic of enquiry to identify, compare and contrast findings and conceptualisations within published literature to begin to address an area of concern. Our initial theory is that conceptualisations of success and failure in the absence of cure influence social processes and interactions for the concurrent delivery of education and health services in the context of multimorbidity in primary care is the reason that we have focused our interest initially within socio-cultural theories. For example, a patient may be prescribed multiple medications for different morbidities. Some of these medications may cause unpleasant side-effects, so that the patient choses to occasionally omit doses if the side-effect is going to limit them in achieving an alternative priority. Such a scenario is often seen, for example with diuretic medication when patients omit doses in the full knowledge of the potential implications in order to undertake social activities without the fear of being unable to reach toilet facilities. Success for the patient means appropriate balancing of symptoms arising from either disease or medication to reduce impact on daily life. Success for professionals may mean compliance with prescriptions or objective measures such as blood pressure control. Success for learners will depend on how patients and professionals introduce and negotiate these different perspectives, relate to each other and achieve shared needs-based goals or not. The outcome of experiencing this and similar situations will impact on learning for future practice in different ways depending on the learner’s prior experience and current need for support to make sense of what they have seen. Social processes are likely to play a significant role in mechanisms that lead to the desired outcome of relationship-centred negotiation to achieve needs-based healthcare and education in this context.

We will, however, be seeking both confirmatory and contradictory findings in order to refine these ideas and any alternative programme theories identified in the literature as we consider the evidence. Results of the review will include description of relevant study characteristics, data identified which supports, refines or challenges theories, new theoretical developments for further empirical research and implications.

We intend to disseminate our work with findings, conclusions and recommendations through presentation and peer-reviewed publications. In addition this work will form the foundation for further research to understand the complex social processes at play within clinical workplaces with a view to informing policy and practice for the development of sustainable healthcare through concurrent education and service delivery for patients with multimorbidity. In doing so we hope to answer the 'so what?’ question that may arise in response to our synthesis in an academically defensible and practically credible way, using the synthesis findings to form a coherent theoretical basis for future research in this area [29].

### Strengths and limitations

While this realist review presents challenges, to achieve a meaningful outcome it is important to (at least attempt to) conduct review work that reflects and is relevant to the complexity of the realities of interactions between patients, learners and practitioners. We anticipate refining and further focusing the review on selected issues, identified as important, as part of the iterative process. To aid transparency of the final review processes we will be using the RAMESES publication standards when reporting both our findings and any changes to the protocol/iterative developments during our conduction of the work [19].

Given that multimorbidity is only a relatively recently investigated issue, and concurrent research about healthcare service provision and education of students is limited, it is possible that our work will identify extensive gaps in the literature. We accept that what we are proposing is to address a complex problem. Simple clear cut studies cannot be undertaken to examine complex problems, however focused research may follow once the important, relevant areas of limited knowledge have been clearly identified. Although it is possible that currently available literature may be insufficient to suggest a full programme theory for future exploration and testing, knowledge of the gaps preventing this is just as crucial to appropriately direct and prioritise further research in under-investigated areas. Should this be the case, then we will instead identify what the shape of new research should be to address such gaps. Given the social nature of education and healthcare it will be important to map the gaps in the literature and assess the current state of understanding of social processes at play with respect to our research questions.

We have also taken the precaution of guarding against unrecognised literature omissions by building a collaborative team across disciplines and perspectives and designing a search strategy which should identify literature relevant to elements of the questions we are seeking to answer, thereby allowing us to synthase a 'bigger picture’ than that gained from any single discipline specific literature review. We accept that the literature may, or may not reflect 'real world practices’ of learners, patients and professionals. This is an example of a specific gap which might be appropriate for further study if our review establishes its existence.

This work has been discussed with a number of clinicians and educators who believe that their attempts to manage the concurrent delivery of healthcare and workplace-based learning can be successful but who also recognise that tensions (despite possible synergies) can arise between 'caring’ and 'training’ citing multimorbidity and illnesses with an absence of cure as examples that can be particularly challenging with respect to both goals. Should it be the case that our current research does not identify similar themes in the literature then further research may be needed to identify and clarify the realities of practice including a richer understanding of exemplar practices demonstrating how challenges can be negotiated to ensure sustainable learning and healthcare delivery.

It is necessary to conduct this synthesis within a particular time and place and we have chosen to contextualise our work within the UK healthcare system. We hope that any programme theory we will develop will contain useful and possibly transferable explanations of the complex interventions used to treat and teach about patients with multimorbidity. However we appreciate that specification of any theory developed for other contexts may well be necessary. We also acknowledge that economics cannot be ignored in future models of medical education and health service delivery. To do justice to this question, however, requires a separate review.

## Endnotes

^a^This statement is based on the following: our review team includes medical students, trainees and doctors who are not aware of any coverage of multimorbidity in their own current undergraduate or postgraduate curricula (as learners and/or teachers), students and trainees who attended discussion groups in the development stage of this protocol raised the absence of learning about, or at least an explicit focus on learning about, multimorbidity as a concern, and searching *Medical Education* and *Academic Medicine*, two of the most eminent journals in the field of medical education for 'multimorbidity’ produced no relevant results. Nor did use of this search term produce any results in published undergraduate/postgraduate curricula standards in the UK.

## Abbreviations

CMO: Context mechanisms outcomes; VICTORE: Volitions implementation contexts time outcomes rivalry emergence; WHO: World Health Organization.

## Competing interests

The authors declare that they have no competing interests.

## Authors’ contributions

SY conceived and designed the study, designed the strategy for systematically identifying relevant literature and recruiting the researcher-participant group of participant-researchers (co-applicants) and steering group (collaborators). She is coordinating the study as principal investigator and wrote the first draft of the study protocol. She contributed to all drafts and finalised the revised version of this manuscript. EC has contributed to the development, reviewing and revising of this protocol and will be a key member of the team analysing the literature. She contributed to all drafts of this manuscript. JP contributed to the design of the study and development of the study protocol. She contributed to intermediate drafts and the final draft of this manuscript. All authors read and approved the final manuscript.

## Authors’ information

SY (BM, PGCertClinEd, MA, PhD, MRCP) is a NIHR Academic Clinical Lecturer in Medical Education at Keele University and Specialist Registrar in Palliative Medicine in the West Midlands Deanery. EC (MBChB, DMedSci, DRCOG, DFSRH) is a NIHR Academic Clinical Fellow in General Practice Specialty Training at Keele University and in the West Midlands Deanery. JP (MB ChB, MRes, PhD, FRCGP) is a Senior Lecturer in General Practice at Keele University and a GP in an inner city practice in Manchester.

## Supplementary Material

Additional file 1 Medline search strategy (via NHS Evidence). Click here for file

Additional file 2 Data extraction sheet (this will be initially piloted as we plan to refine it as our review progresses). Click here for file
